# Impact of revised breakpoints on the categorization of susceptibility of Enterobacterales to temocillin

**DOI:** 10.1093/jacamr/dlad114

**Published:** 2023-11-01

**Authors:** Eric Farfour, Cécile Le Brun, Nicolas Degand, Emeline Riverain, Aurélien Dinh, Thierry Timores, Christel Mamona, Alexandre Vallée, Marc Vasse

**Affiliations:** Service de Biologie Clinique, Hôpital Foch, Suresnes, France; Service de Microbiologie, CHU Bretonneau, Tours, France; Service de Biologie, CH d’Antibes Juan-les-Pins, Antibes, France; Service de Biologie, GH Yvelines Nord, Mantes-la-Jolie, France; Service des Maladies Infectieuses, CHU R. Poincaré, Garches, France; Service de Biologie Clinique, Hôpital Foch, Suresnes, France; Service des Maladies Infectieuses, CHU R. Poincaré, Garches, France; Service d'Epidémiologie-Data-Biostatistiques, Délégation à la Recherche Clinique et à l'Innovation, Hôpital Foch, Suresnes, France; Service de Biologie Clinique, Hôpital Foch, Suresnes, France; UMRS 1176, le Kremlin-Bicêtre, Paris-Saclay, France

## Abstract

**Background:**

To harmonize with the EUCAST breakpoints, the French Society of Microbiology introduced a change in the inhibition diameter breakpoint (17 mm versus 20 mm previously) of temocillin. We assessed the impact of the new breakpoints on categorizing susceptibility of Enterobacterales to temocillin.

**Methods:**

This was a multicentric retrospective study including all Enterobacterales isolates routinely tested for temocillin susceptibility with the disc diffusion method between 1 January 2016 and 31 July 2022 in four centres. Categorization using the breakpoints of 20 mm (French guidelines CA-SFM/EUCAST 2020 v.1.1) and 17 mm (French guidelines CA-SFM/EUCAST 2021 v1.0 and EUCAST guidelines v11.0) was performed.

**Results:**

Overall, 36 416 Enterobacterales isolates were included. The overall rate of temocillin resistance decreased from 11.3% to 4.7% (relative difference of 58.5%) when using the 17 mm breakpoint instead of the 20 mm breakpoint, respectively. The relative change ranged from −44.0% in *Klebsiella aerogenes* to −72.7% in *Klebsiella oxytoca*. The median inhibition diameter was 23 mm (IQR 21–25). The isolates with a diameter of 20 mm appeared overrepresented, whereas those with a diameter of 18 and 19 mm were underrepresented. We therefore reviewed the diameters between 18 and 21 mm of 273 isolates. Thirty-two (11.7%) of them categorized as susceptible at first measure were controlled resistant at second measure.

**Conclusions:**

The new breakpoint induced a decrease in the rate of isolates categorized as resistant to temocillin, increasing therapeutic choice including for Extended-spectrum beta-lactamase-producing Enterobacterales (ESBL-PE). We suggest the bias in measuring the inhibition diameter is probably related to the fact that temocillin is considered remarkably stable against broad-spectrum β-lactamases.

## Introduction

Temocillin is a derivative of ticarcillin that has been synthesized since the early 1980s. Temocillin has a narrow spectrum, and is considered a carbapenem-sparring antibiotic with minimal risk of *Clostridioides difficile* infection.^1,2^ Furthermore, the prevalence of temocillin resistance among Enterobacterales remains low, including in Belgium, where it has been used for more than 30 years.^3–6^

In France, where the drug has been available since 2015, the breakpoints for antibiotic susceptibility testing (AST) were first established at 20 mm for the disc diffusion method and 8 mg/L for MIC determination, whereas they were, respectively, 17 mm and 16 mg/L in Belgium.^7^ To harmonize with the EUCAST v11.0 guidelines,^8^ the breakpoints of the French CA-SFM/EUCAST 2021 v1.0 (Comité de l’Antibiogramme de la Société Française de Microbiologie^9^) guidelines were updated following the introduction of temocillin breakpoints in EUCAST guidelines using the disc diffusion method (17 versus 20 mm previously), and MIC determination (16 versus 8 mg/L).

In the present study, we aimed to assess the impact of the new breakpoints on categorizing the susceptibility of Enterobacterales to temocillin.

## Materials and methods

### Isolates and antibiotic susceptibility testing

Four French centres participated in this retrospective multicentric study. All Enterobacterales isolates recovered from clinical samples and routinely tested for temocillin from 1 January 2016 to 31 August 2022, were included. In all the centres the breakpoint of the French CA-SFM/EUCAST 2019 v1.0 guideline (20 mm) was used for the categorization of Enterobacterales susceptibility to temocillin at the time of inclusion. A single strain displaying the same pattern of resistance per species, year and patient was included. AST was performed using the disc diffusion method according to CA-SFM/EUCAST guidelines in each centre. The inhibition zone diameter of temocillin and three other β-lactams, i.e. amoxicillin, piperacillin/tazobactam and cefotaxime, were recorded as well as the mechanism of resistance to third-generation cephalosporins of non-susceptible isolates. AST was interpreted using the EUCAST v11.0 guidelines for amoxicillin, piperacillin/tazobactam and cefotaxime.^8^ The isolates were categorized for susceptibility to temocillin according to the breakpoint of 20 mm (French CA-SFM/EUCAST 2019 v1.0 guidelines )^9^ and 17 mm (French CA-SFM/EUCAST 2021 v1.0 guidelines and EUCAST v11.0 guidelines).^8,10^ Temocillin breakpoint values are similar in EUCAST v11.0 and French CA-SFM/EUCAST v1.0 guidelines. Nevertheless, in EUCAST they can only be used for *Escherichia coli*, *Klebsiella* spp. (except *K. aerogenes*) and *Proteus mirabilis* originating from infections of the urinary tract. Conversely, in the French CA-SFM/EUCAST guidelines, temocillin breakpoints apply to all Enterobacterales species from all infections. We decided to use this latter definition in the present study. Bacterial identification was performed using conventional biochemical methods or MALDI-TOF MS as recommended by the manufacturers.

### Control of inhibition diameter

Each centre was invited to control the inhibition diameter of 30 consecutive isolates that were first measured between 18 and 21 mm for the following species: *K. aerogenes*, *Serratia marcescens*, *Enterobacter cloacae* complex and *Citrobacter freundii*. The differences between the two measures were recorded and the change of categorization was calculated with the breakpoint of 20 mm.

## Results

**Figure 1.**
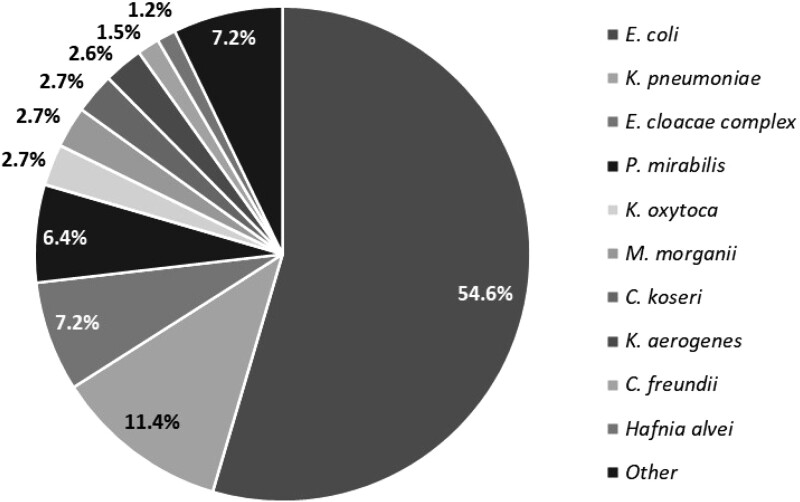
Distribution of bacterial species.

**Figure 2.**
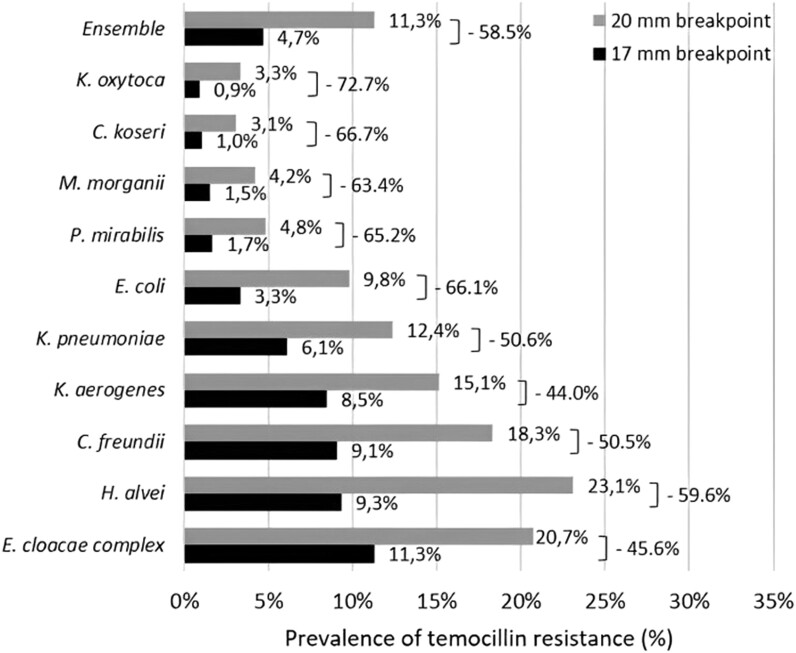
Prevalence of temocillin resistance according to bacterial species.

Overall, 36 416 Enterobacterales isolates were included. The leading species were *E. coli* (54.6%), *Klebsiella pneumoniae* (11.4%) and *E. cloacae* complex (7.2%) (Figure 1). Using the 20 mm breakpoint, the overall prevalence of temocillin resistance was 11.3%. Except for *Morganella morganii*, cephalosporinase-overproducing species displayed the highest rate of temocillin resistance, respectively 43.1%, 20.7%, 18.3% and 15.1% in *S. marcescens*, *E. cloacae* complex, *C. freundii* and *K. aerogenes*. Conversely, the prevalence of temocillin resistance was below 5% in *Klebsiella oxytoca*, *Citrobacter koseri*, *M. morganii* and *P. mirabilis*. The overall rate of strains resistant to temocillin decreased by 58.5% to 4.7% when using the 17 mm breakpoints (Figure 2). The decrease in temocillin resistance ranged from −44.0% in *K. aerogenes* to −72.7% in *K. oxytoca.* The greatest decrease in temocillin resistance occurred in the species in which the prevalence of temocillin resistance was the lowest (i.e. *K. oxytoca*, *C. koseri*, *M. morganii*, *P. mirabilis* and *E. coli*). Using the breakpoint of 20 mm, *E. cloacae* complex and *S. marcescens* were the only species with a prevalence of resistance higher than 10%.

**Figure 3.**
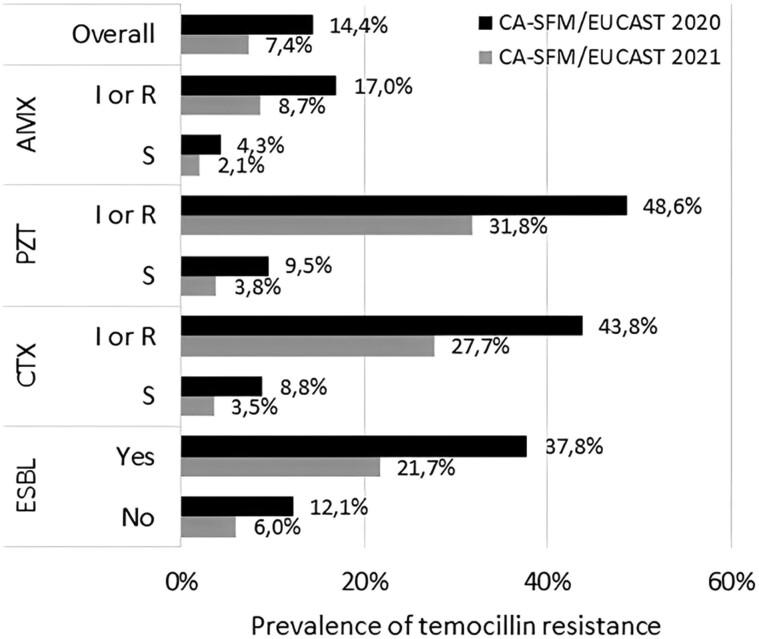
Prevalence of temocillin resistance according to associated resistance to β-lactams: amoxicillin (AMX), piperacillin/tazobactam (PZT), and cefotaxime (CTX). R, resistant; S, susceptible; SDD, Susceptible increased exposure.

The prevalence of temocillin resistance was below 10% in all isolates susceptible to β-lactams using both breakpoints (Figure 3). However, when applying the breakpoint of 20 mm, up to 42.0% and 40.0% of piperacillin/tazobactam-resistant and cefotaxime-resistant isolates were also resistant to temocillin. When using the breakpoint of 17 mm compared with 20 mm, a decrease of 43.6% and 44.3% in the prevalence of temocillin resistance was noted among piperacillin/tazobactam-resistant (prevalence of 23.7%) and cefotaxime-resistant (prevalence of 22.3%) isolates, respectively. This decrease was higher for piperacillin/tazobactam-susceptible (−69.0%) and cefotaxime-susceptible (−65.8%) isolates.

**Figure 4.**
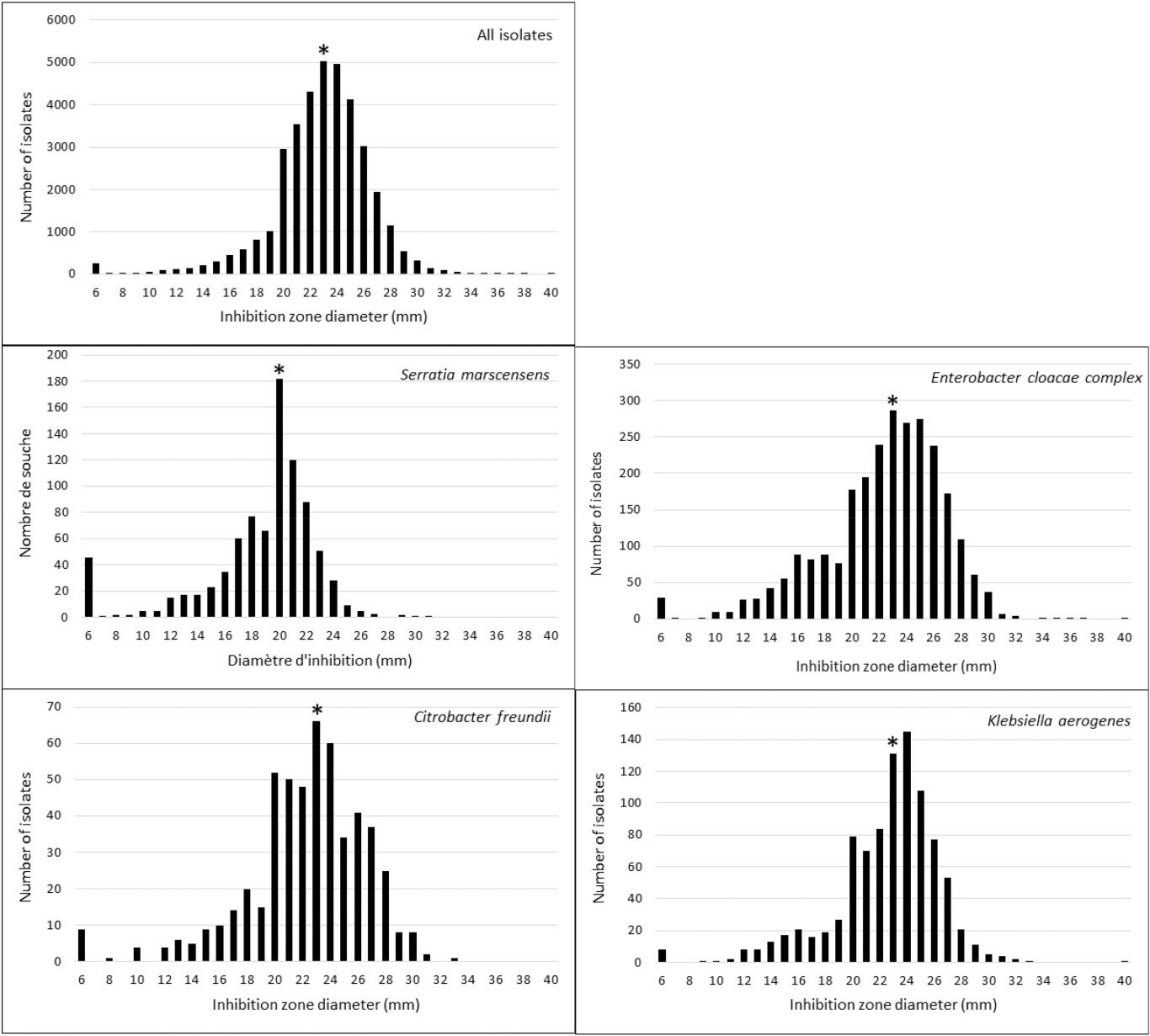

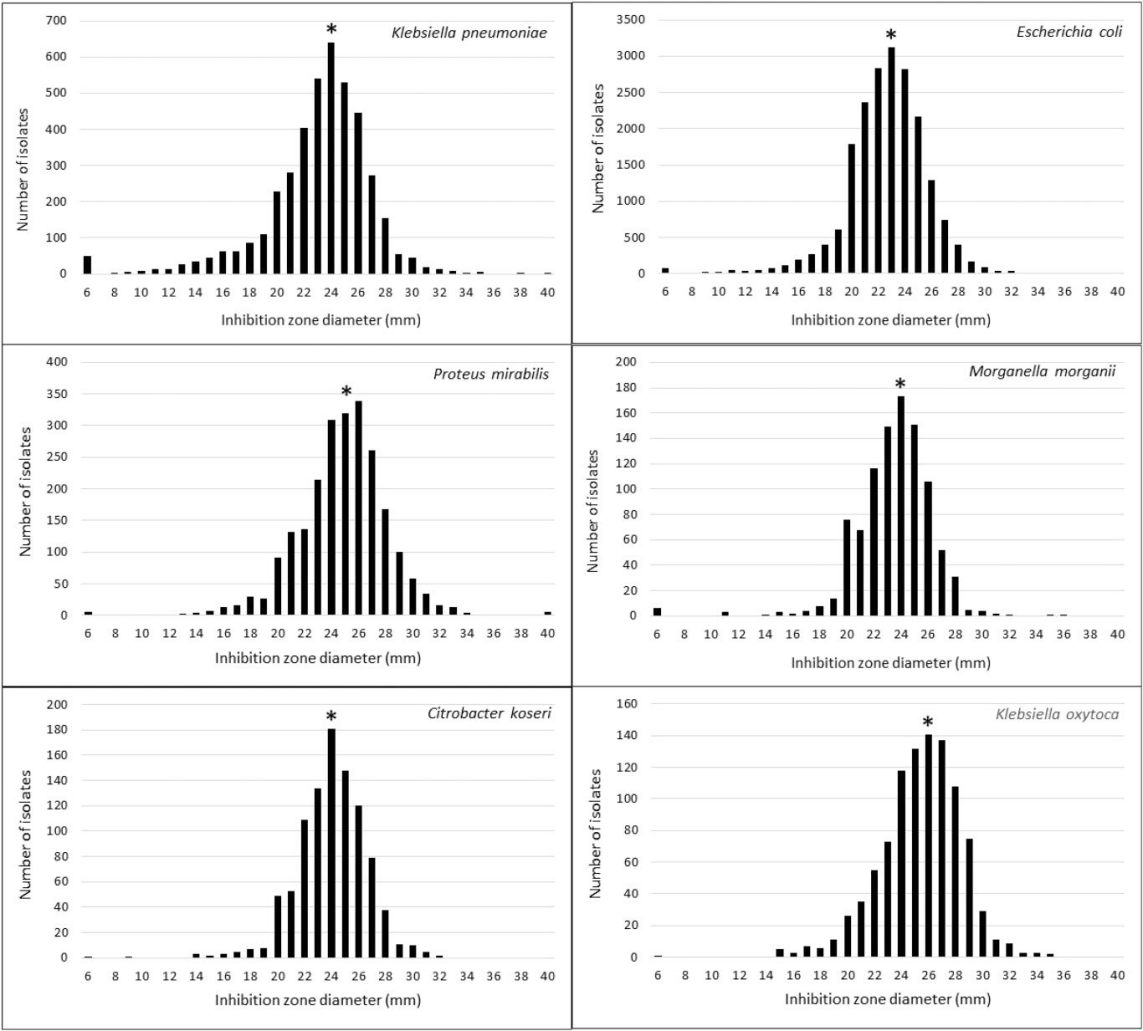
Distribution of inhibition zone diameter of temocillin according to bacterial species.

**Figure 5.**
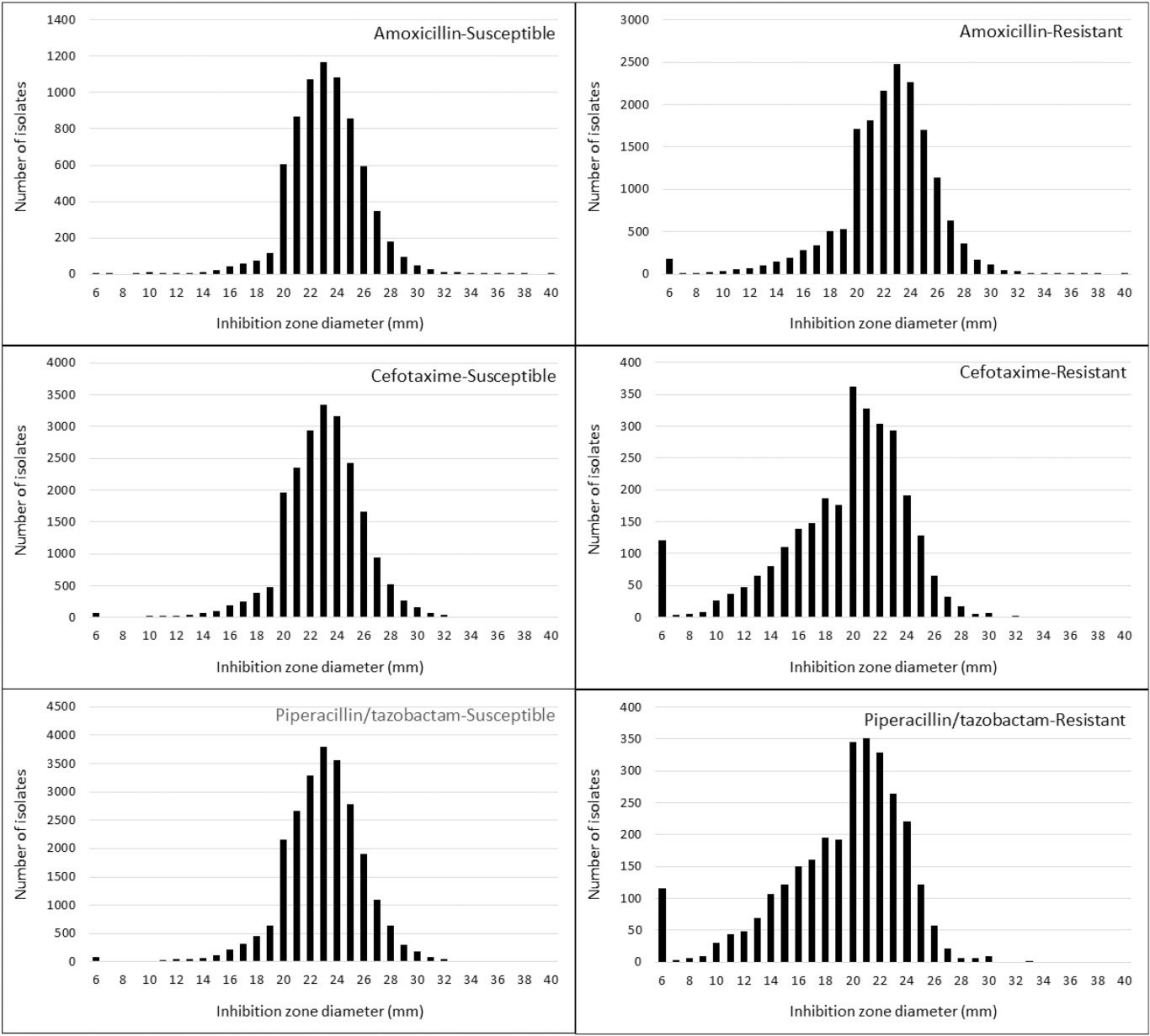
Distribution of temocillin inhibition zone diameter according to susceptibility to amoxicillin, cefotaxime and piperacillin/tazobactam.

The distributions of inhibition diameter according to bacterial species and antimicrobial resistance are represented in Figures 4 and 5. Overall, the median inhibition zone diameter was 23 mm (IQR 21–25). It ranged from 21 mm (IQR 20–23) in *S. marcescens* to 26 mm (IQR 24–27) in *K. oxytoca*. Interestingly, the distribution of the inhibition zone diameter appears imperfectly modal. The number of isolates displaying a diameter of 20 mm appeared higher than expected, whereas those displaying a diameter of 18 and 19 mm were lower. This finding was more marked for the species that have the lower values of inhibition diameter, i.e. *S. marcescens*, *E. cloacae* complex, *C. freundii* and *K. aerogenes*. A similar finding was noted regarding the susceptibility to amoxicillin, cefotaxime and piperacillin/tazobactam. However, the bias in the distribution appeared also more marked for the isolates categorized as resistant to each of these drugs.

We controlled the inhibition diameters of 273 isolates that were first measured between 18 and 21 mm (Table 1.). The second measure was similar in 73.2% of the isolates, and different by 1 mm and at least 2 mm in 19.8% and 7.0%, respectively (Table 1). Five (1.8%) isolates first categorized as resistant to temocillin were controlled susceptible. Conversely, 32 (11.7%) susceptible were controlled resistant to temocillin. Change in categorization mainly concerned *S. marcescens*, in which 23 (23.7%) isolates were categorized resistant instead of susceptible at first measure.

**Table 1.** Impact of second measure of temocillin inhibition diameter in four species

	Number of isolates	Difference of diameter measures (mm)					Change in categorization for a 20 mm diameter	
		≤−2	−1	0	1	≥2	R → S	S → R
*E. cloacae* complex	82	1	6	63	9	3	2 (2.4%)	5 (6.1%)
*C. freundii*	47	0	3	40	3	1	0 (0.0%)	3 (6.4%)
*S. marcescens*	97	1	2	60	25	9	2 (2.1%)	23 (23.7%)
*K. aerogenes*	47	2	1	37	5	2	1 (2.1%)	1 (2.1%)
Overall	273	4	12	200	42	15	5 (1.8%)	32 (11.7%)

R, resistant; S, susceptible.

## Discussion

The change in temocillin breakpoints has led to a decrease in the prevalence of Enterobacterales isolates reported resistant to the drug (4.3% versus 11.4% previously). Nevertheless, the prevalence of temocillin resistance is heterogeneous among bacterial species, ranging from 0.9% in *K. oxytoca* to 19.5% in *S. marcescens*. Moreover, the distribution of the inhibition zone diameter appeared imperfectly modal for diameters ranging from 18 to 21 mm, mainly for cephalosporinase-overproducing species.

The change of breakpoints could impact the categorization of bacterial isolates for a drug. Indeed, as a consequence of the implementation of a breakpoint of 24 mm instead of 19 mm, the overall rate of fosfomycin resistance increased by about 3-fold, from 5.6% to 18.1%.^11^ In France, temocillin is used as a second-line drug as an alternative to broad-spectrum antibiotics, especially for ESBL-producing Enterobacterales in order to reduce the selection pressure of these antibiotics and promote their preservation.^12^ The drug can be used for the documented treatment of complicated urinary tract infections (UTIs), pulmonary infections, bacteraemia and cutaneous infections, but it is mainly used for the treatment of UTIs.^13,14^ In Belgium temocillin is also recommended for the empirical treatment of complicated UTIs.^15^ Empirical treatment for UTIs is recommended according to the antibiotic resistance risk level adapted for the clinical criteria. Accordingly, ≤10% of resistant isolates are required to accept empirical treatment for uncomplicated community-acquired UTIs.^16,17^ We found an overall prevalence of temocillin resistance below 10% when using the new breakpoints, reaching the criterion for the empirical treatment of UTI. However, focusing on clinical isolates recovered from uncomplicated community-acquired UTIs is required to confirm this result. Although breakpoint values for temocillin are similar in EUCAST guidelines and French CA-SFM/EUCAST guidelines, EUCAST guidelines can only be used for *E. coli*, *Klebsiella* spp. (except *K. aerogenes*) and *P. mirabilis*. These differences could be explained by a higher prevalence of temocillin resistance among cephalosporinase-producing Enterobacterales.^4^ There are no differences in the clinical outcome of patients treated with temocillin for infections due to *E. coli*, *K. pneumoniae* and *P. mirabilis*, compared with those involving *E. cloacae* complex or other cephalosporinase-producing strains.^13,18,19^ Nevertheless, a few isolates of cephalosporinase-producing species were included in these studies. It would seem advisable to conduct further studies on infections other than those of the urinary tract, distinguishing the species of Enterobacterales and adapting the dosage.

The distribution of the inhibition zone diameter was singular, with overrepresentation of the number of isolates with a diameter of 20 mm. It could be hypothesized that this distribution is related to the method of AST or a bias in measuring the inhibition diameter. However, the disc diffusion method has good reliability for susceptibility testing of temocillin compared with the broth microdilution and the agar dilution methods, showing only 0.7%–3.3% major error (incorrectly determined as resistant using the disc diffusion method).^20–22^ Furthermore, the bias was more important for the species with the lower values of inhibition zone diameter such as cephalosporinase-overproducing isolates, and those categorized as resistant to at least one β-lactam. The diameter distribution appears modal in bacterial species that display the highest median diameters such as *K. oxytoca* or *C. koseri*. When controlling the inhibition diameter of temocillin, the differences between the two measures were small, ≤1 mm for 93.0% of the isolates, but it led to a change in categorization from susceptible to resistant for 11.7%. Interestingly, these species display the highest value of temocillin MIC.^23^ Overestimation of inhibition diameter is therefore likely due to a bias in measuring. It could be related to the fact that temocillin is considered remarkably stable against broad-spectrum β-lactamases. Indeed, all these isolates were susceptible using the breakpoint of 17 mm.

In conclusion, the harmonization of French breakpoints with European guidelines has led to a decrease in the prevalence of reported temocillin resistance in Enterobacterales. Temocillin resistance in Enterobacterales satisfied the criterion for empirical treatment of complicated UTIs. However, assessing the prevalence of temocillin resistance in these isolates is still required. We suggest that bias in measuring the inhibition diameter is probably related to the fact that temocillin is considered remarkably stable against broad-spectrum β-lactamases.
